# Management of PErioperative Anemia in EmeRgency Laparotomy Patients (PEARL Study)

**DOI:** 10.1002/wjs.70301

**Published:** 2026-03-16

**Authors:** Vignesh Lakshmanan, Dhanya Lakxmi Nantha Kumar, Carven Chin, Alexander H. M. Lukaszewicz, Alun Meggy, Louise M. Silva, Jared Torkington, Julie A. Cornish

**Affiliations:** ^1^ Cardiff and Vale University Health Board (CAVUHB) University Hospital of Wales Cardiff UK; ^2^ Cardiff University Cardiff UK

**Keywords:** anemia, anemia management, discharge planning, emergency laparotomy

## Abstract

**Background/Aim:**

There has been a drive on improving outcomes after Emergency Laparotomy (EmLap) due to the National Emergency Laparotomy Audit (NELA). This has focused mainly on preoperative and intraoperative management with less emphasis on other perioperative aspects. We aimed to assess the prevalence of anemia in EmLap patients and its management in a perioperative period, exploring the gaps in anemia care.

**Methods:**

A retrospective cohort study of prospectively maintained database of 1055 EmLap patients (2016–2019) from a UK tertiary center was performed. Data were extracted from the NELA database, POLO study, electronic records (Welsh Clinical Portal). Statistics were performed in SPSS v27.

**Results:**

Among 740 patients, 77% underwent open surgery, with mean age of 61.9 years (range 18–98), median age of 65 years, and roughly equal sex distribution (female 54%). The median preoperative NELA risk was 3.6% (IQR 1.1–9.8). Over a quarter of patients (28.6%) were anemic on admission. Anemic patients had significantly longer hospital stays (median 12 days; >/ = 11 days, *p* = 0.008) and higher stoma formation rates (54.1% moderate anemia vs. 34.3% nonanemic; *p* = 0.002). Three‐quarters of patients (74.2%) were anemic at discharge (median Hb: 108 g/L, range: 74–129 g/L) but only 12% were treated with oral or IV iron or blood transfusion; only 10% had anemia reported in their discharge letters with appropriate follow‐up and management plan.

**Conclusions:**

Anemia in patients undergoing emergency laparotomy is significantly under‐recognized and inadequately managed at discharge, despite recognized increased morbidity. A structured pathway for continuing anemia treatment and discharge planning is urgently needed to improve outcomes.

## Graphical abstract

There has been a drive on improving outcomes after Emergency Laparotomy (EmLap) due to the National Emergency Laparotomy Audit (NELA). This has focused mainly on preoperative and intraoperative management with less emphasis on other perioperative aspects. We aimed to assess the prevalence of anemia in EmLap patients and its management in perioperative period, exploring the gaps in anemia care.

## 1 Introduction

The prevalence of anemia among patients undergoing major gastrointestinal (GI) surgery is 30%–40%, with even higher rates of up to 80% observed in those undergoing emergency surgery [1, 2, 3]. This condition arises from a complexity of preexisting health issues, surgical blood loss, and postoperative factors that exacerbate baseline anemia [4, 5, 6]. Perioperative anemia in patients undergoing emergency surgery is associated with postoperative complications, prolonged hospital stays, increased likelihood of readmission, and elevated morbidity and mortality [6, 7, 8, 9, 10, 11]. The Center for Perioperative Care (CPOC) supported by the Royal College of Anesthetists (RCoA) emphasizes the increased mortality and morbidity associated with perioperative anemia. They highly recommend that all surgical centers establish anemia pathways spanning the pre, intra‐, and postoperative periods, with special focus on hemoglobin monitoring, documentation at discharge, and coordinated follow‐up with primary care [12].

Recent research underscores the need for systematic screening and management of anemia in surgical patients. A growing body of evidence supports the importance of optimizing preoperative hemoglobin levels to reduce adverse outcomes [11]. Preoperative interventions such as oral or intravenous iron therapy and erythropoiesis‐stimulating agents have shown promise in improving hemoglobin levels and reducing transfusion requirements, thereby mitigating the risks associated with perioperative anemia, which are not feasible during emergency surgery but could be considered postoperatively [13, 14]. Despite national recommendations by the National Institute for Health and Care Excellence (NICE, 2020) and Royal College of Surgeons England (RCSE), the perioperative management and discharge follow‐up of anemia in emergency laparotomy patients remain poorly characterized [15, 16, 17].

This study aimed to identify the incidence of anemia in emergency laparotomy patients during the perioperative period, with a special focus on discharge and the management of anemia. It also aims to develop an understanding of its effect on surgical trajectories and outcomes.

The National Emergency Laparotomy Audit (NELA) was established to improve the quality of care and outcomes for patients undergoing emergency laparotomy in England and Wales. Its primary aims are to monitor and reduce mortality and morbidity associated with EmLap through systematic data collection and benchmarking of perioperative care. Our group previously conducted a study on psychosocial outcomes following emergency laparotomy (POLO study, Silva et al.) at a tertiary center in Wales, United Kingdom (UK) [18]. This study's patient population was derived from the NELA database, POLO study, and additional data collected from patients' medical records.

### 1.1 Aims/Objectives

1. To evaluate the prevalence of anemia at different time points in the perioperative period among EmLap patients:
  - 3 months prior to EmLap,
  - at admission/day of EmLap
  - at discharge, and
  - up to 12 months post‐EmLap.
2. To assess interventions and management of anemia during this period.

## 2 Methods

**FIGURE 1:**
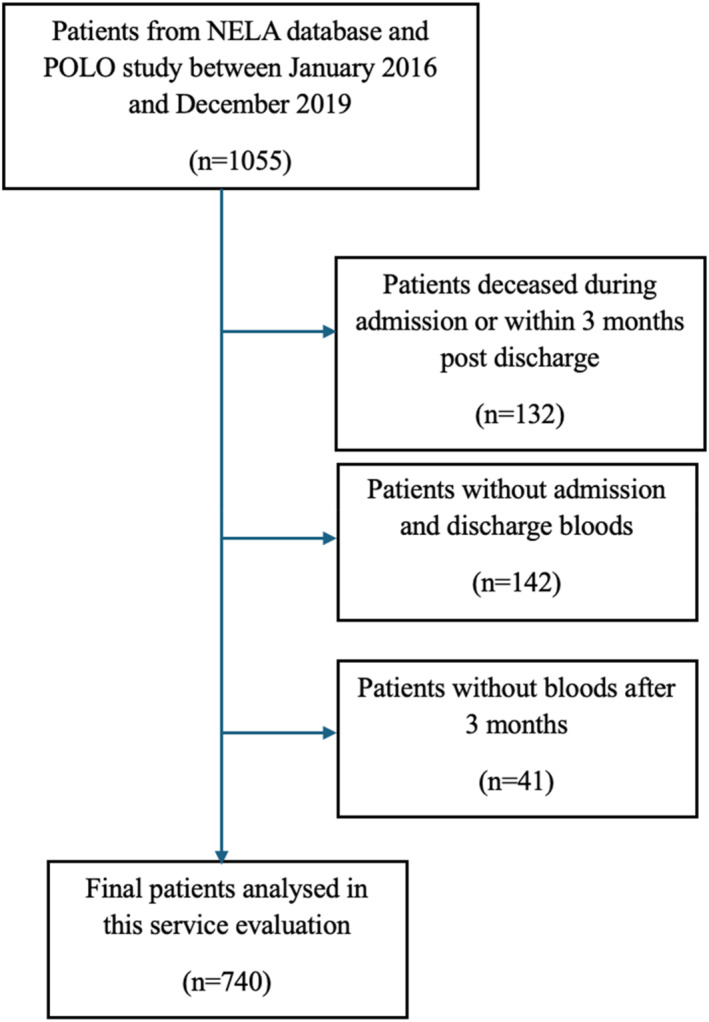
Patient selection consort diagram for the final study cohort.

This was a retrospective cohort study of a prospectively maintained NELA database from the University Hospital of Wales (UHW), a tertiary center in Cardiff, Wales, UK. Patients were identified from the NELA database with additional data from the POLO study, which included all patients who underwent emergency laparotomy between January 2016 and December 2019. A total of 1055 patients underwent emergency laparotomy during that period. Patients who died during the index admission were excluded, as discharge hemoglobin, documentation, and follow‐up planning were not applicable. Including these patients would introduce survivorship bias and outside the scope of this study. Figure 1 shows the patient selection consort diagram for the final study cohort. Electronic medical records using Welsh Clinical Portal (WCP) was used to extract clinical, laboratory, and outcome data. Discharge advice letters (DALs) of all the included patients were analyzed. The data and sources were independently validated by two reviewers (DN, VL) prior to analysis. Data extraction was independently verified to minimize selection and measurement bias. No formal sample size calculation was performed, as all eligible patients with available data within the study period were included.

Anemia was defined as per the World Health Organization (WHO) criteria: Hb < 120 g/L for females and Hb < 130 g/L for males [19]. The severity of anemia was further classified into three categories based on hemoglobin thresholds:

- Mild anemia: hemoglobin 110–129 g/L in men, 110–119 g/L in women
- Moderate anemia: hemoglobin 80–109 g/L in both men and women
- Severe anemia: hemoglobin < 80 g/L in both men and women

For the purpose of this study, we analyzed blood test results obtained within 3 months prior to EmLap, irrespective of the indication for testing. These definitions were applied consistently across the study cohort for classification and analysis.

This manuscript was prepared in accordance with the Strengthening the Reporting of Observational Studies in Epidemiology (STROBE) guidelines for observational studies [20].

There was no use of Artificial Intelligence Generated Content (AIGC) tools such as ChatGPT and others based on large language models (LLMs) used in developing any portion of their manuscript.

### 2.1 Statistical Analysis

All analyses were performed using IBM SPSS Statistics version 27. The primary aim of this study was to describe the prevalence, recognition, and treatment of perioperative anemia. The study was undertaken as a service evaluation of care processes rather than an outcome prediction study. Therefore, multivariable analysis was not performed. Comparisons between anemia severity groups and postoperative outcomes were assessed using chi‐square tests for univariate analysis, with descriptive statistics presented where appropriate.

### 2.2 Results

A total of 740 patients were included in the final analysis. Median age was 65 (IQR: 49–75.5), and median BMI was 26.3 (IQR: 23–31.29). The median preoperative NELA risk was 3.6% (IQR: 1.1–9.8), and most patients were aged ≥ 70 years (40.4%), with 54.6% being female.

Demographics of the cohort and operative details are provided in Table 1. Most procedures were performed via an open approach (77%), whereas 15.9% were laparoscopic, and 7.2% were laparoscopic converted to open. A total of 19.5% of patients had inflammatory bowel disease. Malignancy was present in 21.6% of patients, where 16.4% had a new diagnosis, and 5.2% had preexisting malignancy. 675 patients had available blood results from 3 months prior to EmLap, and 27.1% of them were anemic.

**TABLE 1:** Patient demographics and relevant clinical details.

Continuous variables	Total (*n* = 740)
	Median (IQR)
Age (years)	65 (49–75.5)
BMI	26.3 (23–31.29)
Preop NELA risk (%)	3.6 (1.1–9.8)

Categorical variables	*n* (%)
Age category	
18–29	35 (4.7)
30–49	154 (20.8)
50–69	252 (34.1)
≥ 70	299 (40.4)
Sex	
Male	336 (45.4)
Female	404 (54.6)
M:F ratio	1:1.2
Surgical approach	
Open	570 (77)
Laparoscopic	118 (15.9)
Lap converted to open	52 (7.2)
3 months prior to EmLap (missing data, *n* = 65)	
No anemia	492 (72.9)
Anemia	183 (27.1)
ASA grading	
I	82 (11.1)
II	285 (38.5)
III	288 (38.9)
IV	79 (10.7)
V	6 (0.8)

### 2.3 Postoperative Outcomes

During index admission, the overall surgical site infection (SSI) rate was 4.2% and was highest in patients with severe anemia (14.3%). Cardiovascular complications were uncommon overall but were observed more frequently in patients with moderate (6.0%) and severe anemia (14.3%) compared with those without anemia (0.9%). Other complications, including urinary tract infections (UTIs) (1.2%), deep vein thrombosis (DVT)/pulmonary embolism (PE) (0.5%), and bleeding (0.7%), were uncommon during index admission. Stoma was formed in 37.2% of patients and occurred more frequently in anemic patients, particularly in the moderate (54.8%) and severe (21.4%) groups. Anemia severity at admission was associated with stoma formation (*χ* ^2^ = 14.778, df = 3, *p* = 0.002). Median length of stay (LOS) was 12 days (IQR: 7–21). When LOS was categorized (0–5 days, 6–10 days, and ≥ 11 days), anemia severity was associated with prolonged hospitalization (*χ* ^2^ = 17.249, df = 6, *p* = 0.008). LOS exceeded 11 days in 54.9% of cases and increased with anemia severity (71.4% in severe anemia vs. 51.4% in nonanemic patients). These comparisons are unadjusted and should be interpreted descriptively.

### 2.4 Prevalence and Severity of Anemia

**FIGURE 2:**
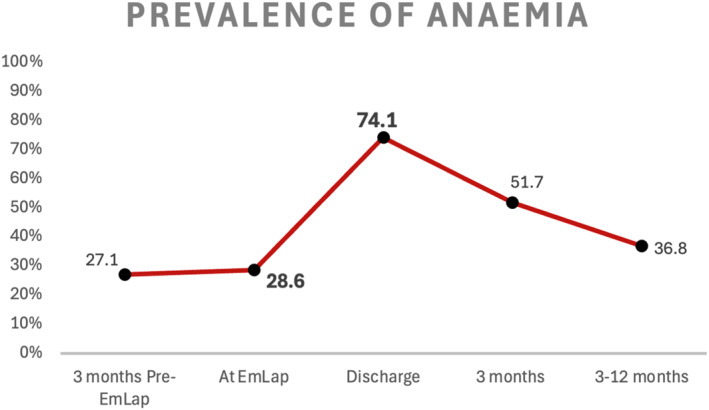
Percentage (%) prevalence of anemia at different time points.

Figure 2 shows the percentage (%) prevalence of anemia at different time points in relation to the day of EmLap. Over a quarter of patients (27.1%) were anemic from 3 months prior to EmLap, with slight increase to 28.6% on the day of EmLap (admission). There is a steep rise to 74.2% of patients being anemic at discharge.

Postoperatively, more than half of the patients (51.5%) were still anemic at 3 months, and 36.8% were anemic up to 12 months after EmLap. Normocytic anemia was the most common subtype across all phases. Among the 212 anemic patients on the day of the EmLap, 114 patients (53.8%) were persistently anemic at 12 months after the EmLap.

Table 2 shows the prevalence of anemia by sex. In terms of severity, on the day of EmLap, mild anemia was observed in 9.9% of patients, which was more common in females (12.1%) than in males (7.1%). Moderate anemia was noted in 11.5% of the participants, which was higher in females (12.9%) than in males (9.8%). Severe anemia was uncommon (1.9%), with a similar sex distribution. By discharge, moderate anemia became the most prevalent type (41.8%), affecting 48.3% of females and 33.9% of males.

**TABLE 2:** Prevalence and types of anemia by sex.

Time point & anemia status	Total *n* (valid %)	Male *n* (valid %)	Female *n* (valid %)
Day of EmLap (*n* = 740)			
Anemic	212 (28.6)	105 (31.3)	107 (26.4)
Nonanemic	528 (71.4)	230 (68.7)	298 (73.6)
Discharge (*n* = 740)			
Anemic	549 (74.2)	240 (71.6)	309 (76.3)
Nonanemic	191 (25.8)	95 (28.4)	96 (23.7)
3 months post‐op (*n* = 268)			
Anemic	139 (51.7)	73 (58.9)	66 (45.5)
Nonanemic	130 (48.5)	51 (41.1)	79 (54.9)
3–12 months post‐op (*n* = 679)			
Anemic	250 (36.8)	109 (36.1)	141 (37.4)
Nonanemic	429 (63.2)	193 (63.9)	236 (62.6)

### 2.5 Anemic Treatment and Discharge Management

Among the 28.6% who were anemic on the day of EmLap, 31 patients received oral iron (median Hb: 107 g/L, range: 83–128 g/L), comprising 14 with mild anemia and 17 with moderate anemia. 19 patients received blood transfusion (median Hb: 101 g/L, range: 53–125 g/L), including 7 with severe anemia, 6 with mild anemia, and 4 with moderate anemia. Four patients were managed with a combination of oral and IV iron (median Hb: 107 g/L, range: 79–124 g/L); among them, two had mild anemia, one had moderate anemia, and one had severe anemia. The remaining 158 patients did not receive any treatment for anemia (median Hb: 111 g/L, range: 42–130 g/L).

At hospital discharge, 549 patients (74.2%) remained anemic. Among these, 43 received oral iron (median Hb: 100 g/L, range: 69–127 g/L), of whom 33 had moderate anemia, eight had mild anemia, and one had severe anemia. 19 patients received blood transfusion (median Hb: 102 g/L, range: 54–117 g/L), including 10 with moderate anemia, 5 with mild anemia, and 4 with severe anemia. Five patients were treated with both oral and IV iron (median Hb: 91 g/L, range: 84–109 g/L), all of whom had moderate anemia. 483 patients were discharged without any treatment for anemia, with a median Hb of 108 g/L (range: 74–129 g/L).

Analysis of discharge advice letters (DALs) of all patients revealed that although anemia prevalence was 74.2% at discharge, only 12% were treated, and only 10% had anemia reported in their discharge letters with appropriate follow‐up and management plans.

## 3 Discussion

In this study of 740 patients who underwent emergency laparotomy, 3‐quarters of the patients (74.2%) were anemic at hospital discharge, and half remained anemic at 3 months postoperatively. Only 12% of the patients received active treatment, and communication to primary care regarding anemia management was documented in only 10% of the patients. Discharge letters mainly included presenting complaint, surgical details (procedure name, date, and indication), and discharge medication lists. Significant aspects of the clinical course during admission, the rationale for commencing new medications, new‐onset anemia, hemoglobin values, cause of anemia, and follow‐up advice or appointments were rarely documented. Iron supplementation plans and GP monitoring instructions were essentially absent. This highlights a major gap in the recognition, communication, and ongoing management of anemia in this high‐risk surgical cohort.

This study is limited by its retrospective design and reliance on paper records, which restricts data completeness. Complications were assessed only during the index admission, likely underestimating post‐discharge morbidity, for example, surgical site infections, which occurred in 4.2% of our cohort compared with 30%–40%, were reported in the literature [21]. The absence of multivariable adjustment limits inference regarding independent associations between anemia and outcomes such as complications or LOS. Confounding by age, comorbidity and surgical indication is possible. However, this does not affect the primary objectives of this study. Future work aims to incorporate prospective, longitudinal data collection, and multivariate analysis to address these gaps.

Anemia is an independent predictor of poor surgical outcomes, including increased complications and mortality [6, 21, 22]. In our study, anemic patients had a greater incidence of cardiovascular and thromboembolic events, longer hospital stays, and increased stoma formations than nonanemic patients, which is consistent with previous reports [13, 23, 24]. There is national guidance and evidence supporting the use of postoperative IV iron, oral iron supplementation, and targeted workup for underlying causes (e.g., malignancy, chronic inflammation, nutritional deficiencies) to mitigate the longer‐term risks of anemia [14, 23, 25]. These interventions are most effective when initiated early, but there is no clear discharge advice or communication to primary care for postoperative anemia follow‐up and management. This suggests that anemia is often perceived as a secondary or transient issue in the acute setting rather than a modifiable risk factor that warrants targeted follow‐up. Emergency surgery patients frequently transition back to community care after discharge; therefore, effective handover to general practitioners (GPs) through discharge information is essential and poses a missed opportunity risk factor as evident from this study.

The NICE framework recommends adopting enhanced recovery pathways in emergency surgeries like those used in elective settings, and RCSE stresses on improving discharge communication to ensure safer transitions of care, particularly for high‐risk patients [16, 17]. Despite such recommendations, anemia at discharge remains widely under‐recognized. Large scale multicenter studies from the United Kingdom (Kanga et al.), Australia (Makar et al.), the United States of America (Warner et al.), and Singapore (Abdullah et al.) consistently report poor post‐discharge management, higher readmission rates, and impaired quality of life among anemic surgical patients [10, 11, 15, 16]. The POSTVenTT Collaborative across Australia and New Zealand similarly identified post‐discharge anemia as a missed therapeutic opportunity [26].

Large studies link anemia to fatigue, delayed recovery, reduced quality of life, readmissions, and cardiovascular complications and the impact extends beyond laboratory thresholds alone [10, 15, 24]. Despite national guidance and international evidence, in our cohort, only 12% of anemic patients received active treatment during admission or at discharge, and only 10% had anemia reported in their discharge letters with appropriate follow‐up and management plans. No formal perioperative anemia pathway existed locally during the study period between 2016–2019, and management choices, whether oral iron, intravenous iron, or blood transfusion, are inconsistent and not guided by anemia severity. Some patients with mild anemia received both oral and intravenous iron, whereas others with moderate or severe anemia received none. This suggests that treatment decisions are not based on the severity of anemia and neither followed the standardized criteria or protocol.

There is currently an NHS Wales Postoperative Anemia Pathway developed by the Blood Health National Oversight Group (BNHOG), which provides a structured, severity‐based guideline and was not in practice during the study period [27]. Anemia identification should be followed by a series of investigations, including hematinics and iron studies, and a follow‐up full blood count in primary care at 4 weeks post‐discharge. This is particularly important in patients with new‐onset moderate to severe anemia, as it may allow identification of persistent anemia and guide treatment or management, in line with national perioperative anemia guidance [12].

Given the substantial undermanagement and inconsistent treatment of anemia demonstrated in this study, adherence to the NHS Wales Postoperative Anemia Pathway is essential to standardize care, improve follow‐up, and reduce avoidable postoperative morbidity in EmLap patients.

## 4 Conclusion

Postoperative anemia after emergency laparotomy is common, under‐recognized, and associated with long‐term adverse outcomes. The implementation of clear management strategies, adherence to NHS Wales Postoperative Anemia pathway, and communication between primary and secondary care are needed.

## Author Contributions

**Vignesh Lakshmanan:** conceptualization, methodology, investigation, validation, data curation, formal analysis, writing – original draft, writing – review and editing. **Dhanya Lakxmi Nantha Kumar:** conceptualization, methodology, investigation, validation, writing ‐ original draft, writing – review and editing. **Carven Chin:** investigation, writing – review and editing. **Alexander H. M. Lukaszewicz:** investigation, writing – review and editing. **Louise M. Silva:** investigation, writing – review and editing. **Alun Meggy:** writing – review and editing. **Jared Torkington:** supervision, writing – review and editing. **Julie A. Cornish:** conceptualization, methodology, supervision, writing – review and editing.

## Funding

The authors have nothing to report.

## Ethics Statement

This study was reviewed by the local Research & Development Department, which determined that ethical approval was not required and was granted approval as a service evaluation.

## Conflicts of Interest

The authors declare no conflicts of interest.

## Data Availability

The data that support the findings of this study are available from the corresponding author upon reasonable request.
